# Blastic Plasmacytoid Dendritic Cell Neoplasm: A Case Series and Review of Literature

**DOI:** 10.7759/cureus.93784

**Published:** 2025-10-03

**Authors:** Shula Staessens, Nathan De Beule, Fabienne Trullemans, Ann De Becker

**Affiliations:** 1 Department of Clinical Hematology, Universitair Ziekenhuis Brussel, Jette, BEL

**Keywords:** blastic plasmacytoid dendritic cell neoplasm (bpdcn), bpdcn, hyper-cvad, tagraxofusp, venetoclax

## Abstract

Blastic plasmacytoid dendritic cell neoplasia (BPDCN) is a rare hematological malignancy. The heterogeneous presentation of this disease can make it difficult to diagnose. Another challenge presents itself when selecting the appropriate treatment as there are currently no treatment guidelines. We present three cases of BPDCN, each treated with a different treatment modality. The first-line treatment in one patient was tagraxofusp, followed by allogeneic stem cell transplant. The second patient was treated with venetoclax in monotherapy. The third patient was treated with a combination of venetoclax and intensive chemotherapy followed by allogeneic stem cell transplant as a consolidating therapy.

## Introduction

Blastic plasmacytoid dendritic cell neoplasia is a rare disease with an incidence of around 0.03-0.04 in 100,000 people [1]. It has a poor prognosis if left untreated. The median overall survival in all age groups combined is 12-24 months [2]. It often presents with bone marrow and lymph node infiltration, but it is also commonly associated with extramedullary disease such as skin lesions and central nervous system (CNS) involvement. Systemic disease with bone marrow, blood, lymph node and/or CNS involvement occurs in about 50% of patients. Depending on whether patients are eligible for intensive treatment or not, multiple options have been proposed. Treatments consist of chemotherapy, immunotherapy and stem cell transplantation [1]. At present, there is only one approved CD123-targeted therapy with tagraxofusp. It was approved by the Food and Drug Administration (FDA) in 2018 [3] and received European Medicines Agency (EMA) marketing authorization for first-line treatment in 2021 [4]. But with increased research and renewed attention after being classified as a separate entity in the World Health Organization (WHO) 2008 classification, new treatment strategies are being investigated both with new agents as well as combinations of existing therapies [1,5]. We present three cases of BPDCN. Two patients were eligible for intensive treatment followed by allogeneic stem cell transplant (alloHSCT) and one patient was considered to be ineligible for intensive treatment and received venetoclax in monotherapy. Only one of our patients received tagraxofusp via medical need program as there is no reimbursement for this treatment in Belgium.

## Case presentation

Case 1

**Figure 1:**
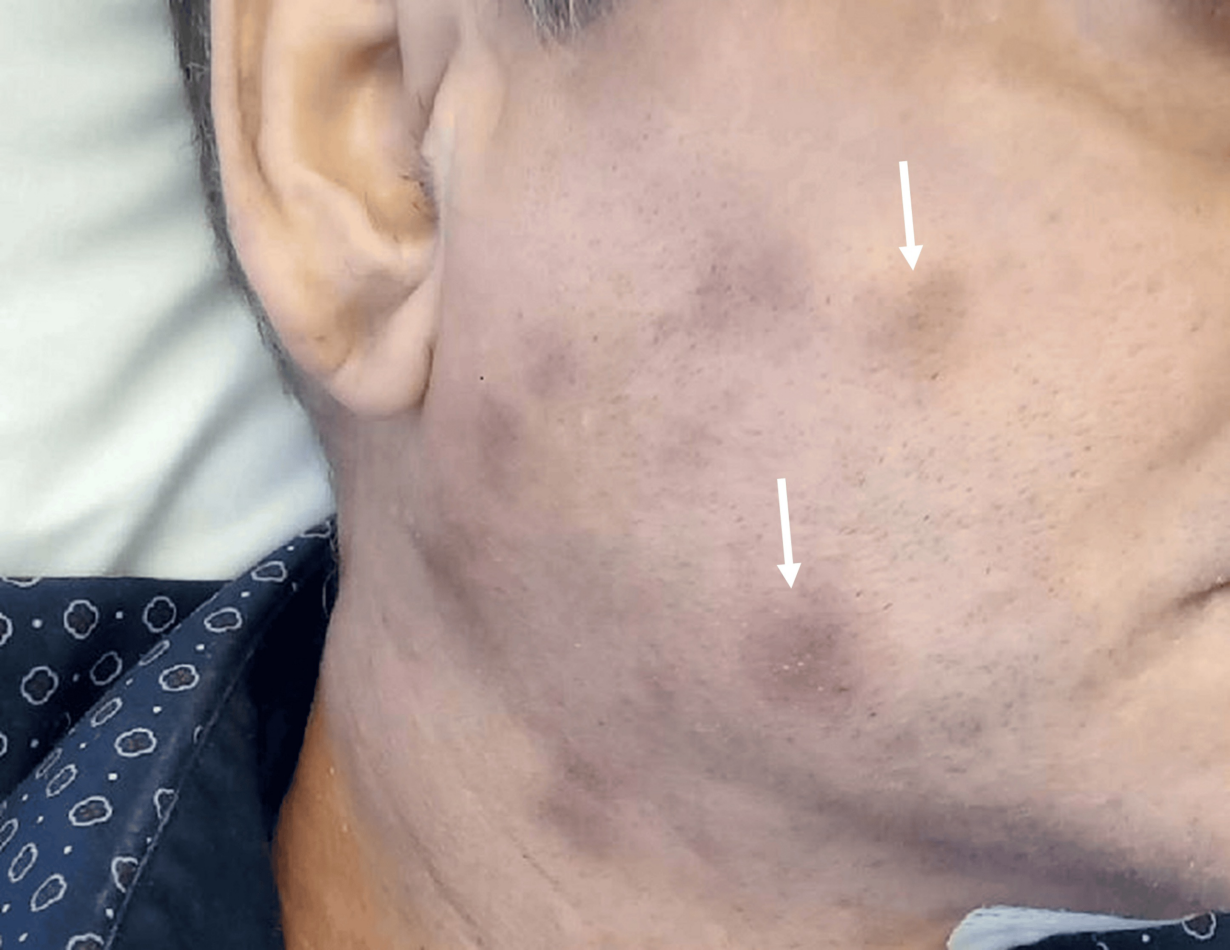
Skin lesions in patient 1 with blastic plasmacytoid dendritic cell neoplasm Macular, bruise-like skin lesions on the face (white arrows)

A 60-year-old male patient presented in December 2022 with weight loss, brown macular skin lesions (Figure 1), anemia and leukocytosis with a peripheral blast count of 62%. In August 2022, he had previously been diagnosed with a myelodysplastic syndrome, refractory anemia with ring sideroblasts (MDS-RARS). Laboratory findings are shown in Table 1.

**Table 1:** Biochemistry analysis of patient 1 on admission

Parameter	Value	Reference
Creatinin	0.92 mg/dL	0.67-1.17 mg/dL
Estimated glomerular filtration rate (eGFR)	89 ml/min/1.73 m²	>60 ml/min/1.73 m²
Albumin	38 g/L	35-50 g/L
C-reactive-protein (CRP)	24.3 mg/L	<5 mg/L
Lactate dehydrogenase	1471 U/L	313-618 U/L
Hemoglobin	6.4 g/dL	11.8-14.5 g/dL
Thrombocytes	55x10³/mm³	158-450x10³/mm³
Leucocytes	16.2x10³/mm³	3.6-9.6x10³/mm³
Blast (absolute count)	10.287x10³/mm³	0x10³/mm³

**Figure 2:**
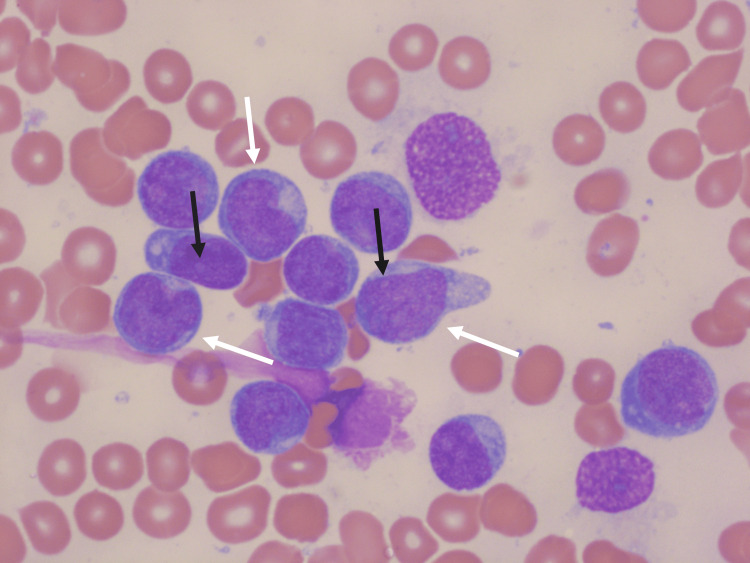
Microscopic analysis of the bone marrow aspirate of patient 1 Large blastic cells with a high nucleus/cytoplasm ratio (white arrows) with often the presence of a nucleolus (black arrows). May-Grünwald Giemsa staining, original magnification 100x.

Bone marrow aspirate was performed and confirmed the diagnosis of BPDCN with the following immunophenotype: CD123++/CD4+/CD56+/HLA-DR++/CD304+/CD45RA+/CD43+/CD36+. Microscopic findings of the bone marrow are shown in Figure 2. Next-generation sequencing (NGS) showed a pathogenic missense variant in SF3B1 and a stop-gain variant in TET2. Karyotype was abnormal and showed trisomy 8.

Lumbar puncture showed signs of CNS involvement, and a population with the same immunophenotype as described above was found. A skin biopsy of a skin lesion on the cheek was positive for BPDCN as well. A computed tomography (CT) scan showed cervical, axillary, mediastinal, and mesenterial lymph nodes, which were not biopsied. Tagraxofusp was given in combination with triple intrathecal chemotherapy (methotrexate (MTX), cytarabine, and methylprednisolone). There was a fast clearance of CNS disease after only one cycle of intrathecal chemotherapy. Tagraxofusp was given for four cycles after which consolidation with an allogenic stem cell transplantation with a matched unrelated donor was performed. Our patient did not develop capillary leak syndrome. Albumin was prophylactically given according to the product information provided by the manufacturer when serum albumin is <3.5 g/L or when serum albumin decreases more than 0.5 g/L below the value at the start of the cycle [4]. Two years after allogeneic stem cell transplantation, the patient fares well and is in complete remission.

Case 2

**Figure 3:**
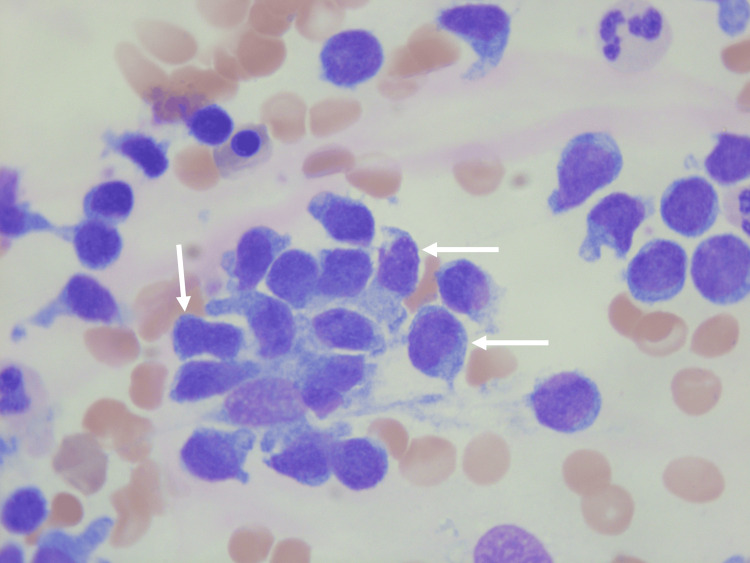
Microscopic analysis of the bone marrow aspirate of patient 2 More mature lymphoid-like abberant cells with an irregular cytoplasm (white arrows) May-Grünwald Giemsa staining, original magnification 100x

A 82-year-old female patient presented in November 2024 with weight loss and pancytopenia. Laboratory analysis is shown in Table 2. A bone marrow aspirate was performed and based on the following immunophenotype: CD123++/CD4+/CD56+/HLA-DR++/CD303+/CD304+/CD45RA++, and a diagnosis of BPDCN was made. Microscopic findings of the bone marrow are shown in Figure 3. NGS showed no variants and a complex karyotype was found. A lumbar puncture was not performed in this patient. Given the advanced age, the presence of CNS involvement would not have had therapeutic consequences. There were no skin lesions. A CT scan showed no signs of lymphadenopathy nor hepatosplenomegaly.

**Table 2:** Biochemistry analysis of patient 2 on admission

Parameter	Value	Reference
Creatinin	0.94 mg/dL	0.67-1.17 mg/dL
Estimated glomerular filtration rate (eGFR)	61 ml/min/1.73 m²	>60 ml/min/1.73 m²
Albumin	38 g/L	35-50 g/L
C-reactive-protein (CRP)	18.1 mg/L	<5 mg/L
Lactate dehydrogenase	159 U/L	313-618 U/L
Hemoglobin	10.0 g/dL	11.8-14.5 g/dL
Thrombocytes	119x10³/mm³	158-450x10³/mm³
Leucocytes	2.3 x 10³/mm³	3.6-9.6x10³/mm³
Blast (absolute count)	0x10³/mm³	0x10³/mm³

In this elderly and frail patient, intensive chemotherapy was not an option. The medical need program for tagraxofusp was closed and it was no longer available for this patient. We then opted for treatment with venetoclax (BCL2 inhibitor) monotherapy for the mitigation of disease progression and alleviation of disease symptoms. An individualized ramping up schedule with a targeting dose of 200 mg was used. The patient received 50 mg every two days for a week, 50 mg daily for a week, and 100 mg daily for a week. Antimicrobial prophylaxis was added in the form of fluconazol; therefore, the dose of venetoclax was kept at 100 mg daily.

A reevaluation of the bone marrow aspirate in June 2025, after four cycles of treatment, showed a good response with the presence of only 0.3% of leukemic cells, compatible with minimal residual disease. No full hematological recovery was obtained, which was attributed to venetoclax-related myelosuppression. This was mitigated with transfusion and granulocyte colony-stimulating factor (G-CSF). The patient's general condition had improved during treatment.

Case 3

**Figure 4:**
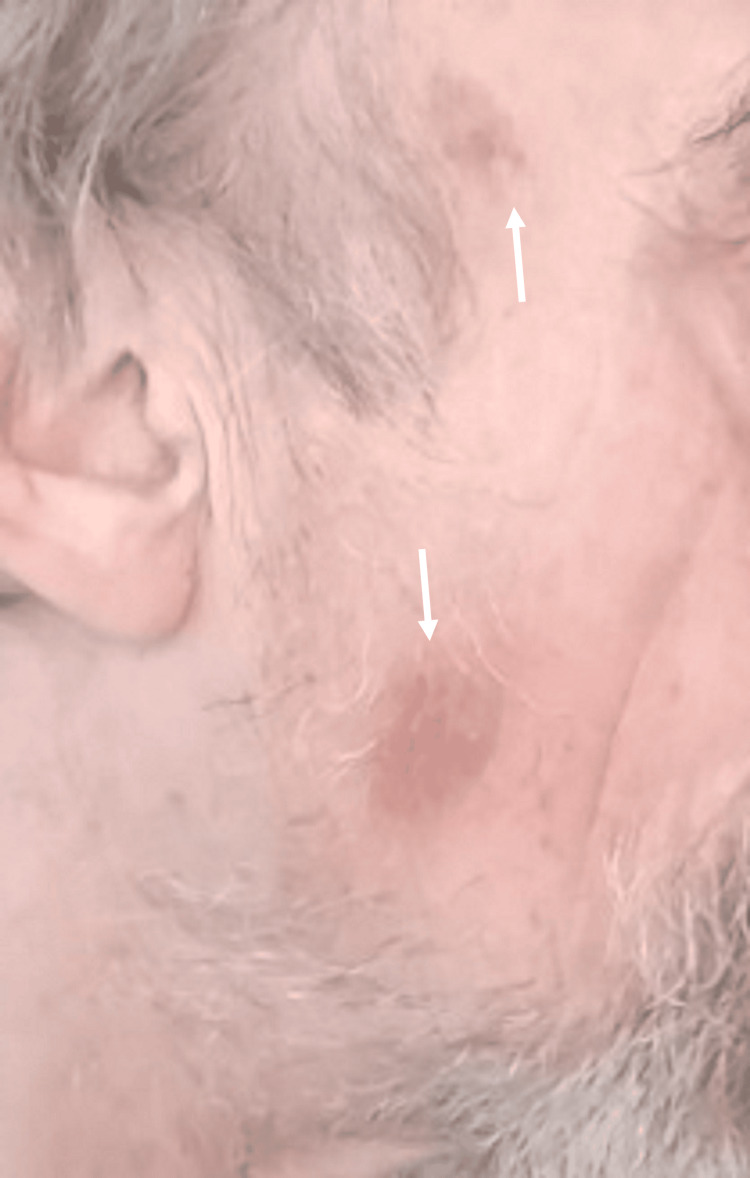
Skin lesions in patient 3 with blastic plasmacytoid dendritic cell neoplasm Macular, bruise-like skin lesions on the face (white arrows)

In November 2024, a 67-year-old male patient presented with reddish-brown, macular skin lesions on the face, arms and thorax since a few months (Figure 4). Biochemistry analysis on admission is shown in Table 3.

**Table 3:** Biochemistry analysis of patient 3 on admission

Parameter	Value	Reference
Creatinin	1.11 mg/dL	0.67-1.17 mg/dL
Estimated glomerular filtration rate (eGFR)	65 ml/min/1.73 m²	>60 ml/min/1.73 m²
Albumin	37 g/L	35-50 g/L
C-reactive-protein (CRP)	8.3 mg/L	<5 mg/L
Lactate dehydrogenase	3909 U/L	313-618 U/L
Hemoglobin	13.4 g/dL	11.8-14.5 g/dL
Thrombocytes	90x10³/mm³	158-450x10³/mm³
Leucocytes	8.9x10³/mm³	3.6-9.6x10³/mm³
Blast (absolute count)	1.420x10³/mm³	0x10³/mm³

**Figure 5:**
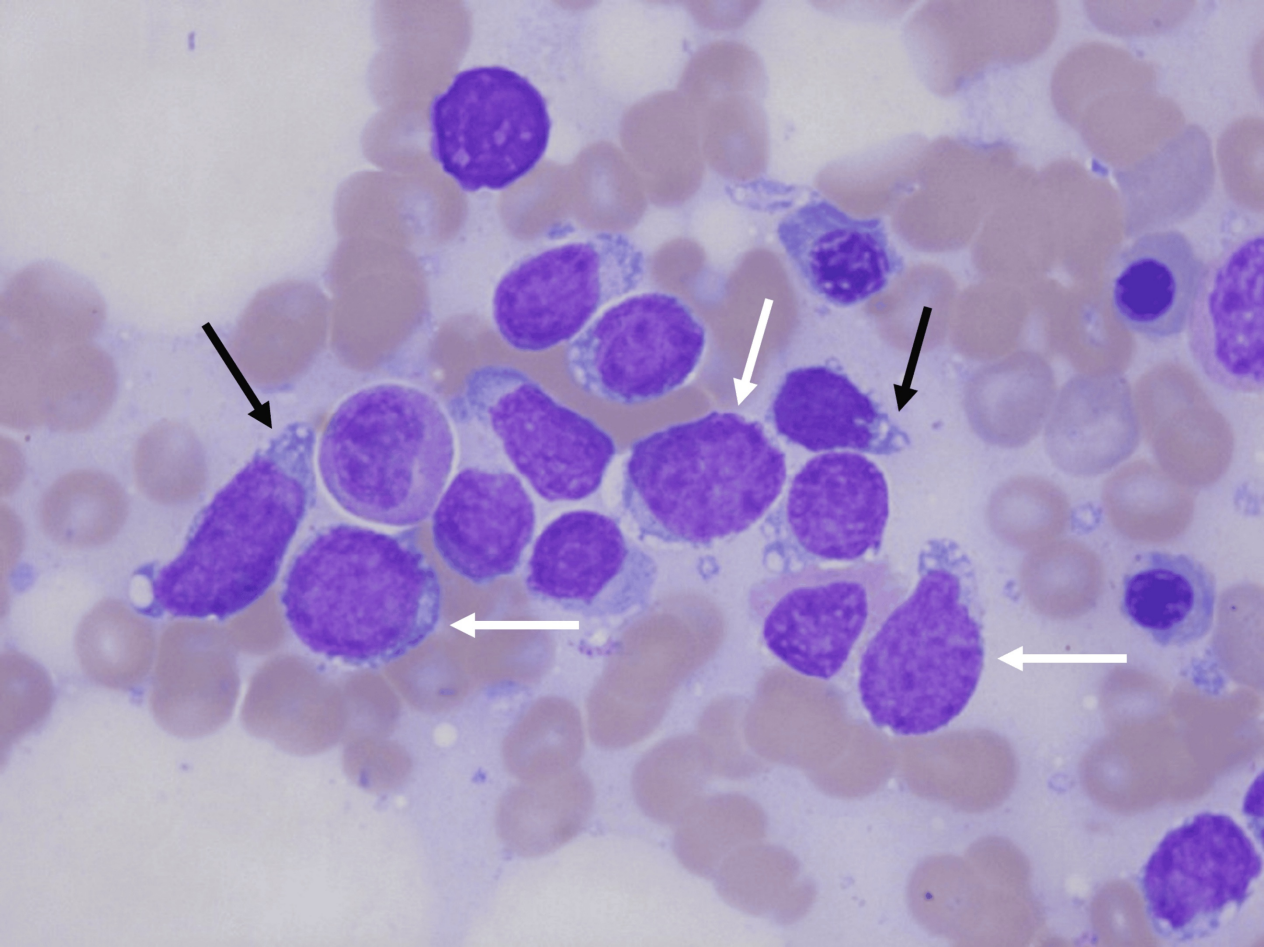
Microscopic analysis of the bone marrow aspirate of patient 3 Blast-like cells with a high nucleus/cytoplasm ratio (white arrows), some of which with pseudopodia (black arrows). May-Grünwald Giemsa staining, original magnification 100x.

The diagnosis of BPDCN was confirmed on bone marrow biopsy, revealing massive infiltration (around 90%) by cells with the following immunophenotype: CD123++/CD4+/CD56+/HLA-DR++/CD303-/CD304 +/CD45RA+. Microscopic findings of the bone marrow are shown in Figure 5. NGS showed a pathogenic frameshift and stop gain variant in TET2 and a pathogenic frameshift in ASXL1 and the karyotype was normal.

A biopsy of the skin lesions showed a localization of BPDCN and a lumbar puncture excluded CNS invasion. Positron emission tomography (PET) scan showed a few small, hypermetabolic axillary and inguinal lymph nodes, which were not biopsied. The medical need program for tagraxofusp had been closed and it was no longer available for our patient. This patient, however, was eligible for treatment with intensive chemotherapy. He received Hyper-CVAD [6] in combination with venetoclax and CNS prophylaxis with intrathecal chemotherapy (MTX and Cytarabine). The first cycle venetoclax was given for seven days using a ramping up schedule. The patient received 100 mg on day one, 200 mg on day two and 400 mg from day three to seven. From cycle two to cycle four, the patient received venetoclax 400 mg from day one to day three. After four cycles (cycle one and three: part A, cycle two and four: part B), complete remission was obtained and an allogenic stem cell transplant with a matched related donor was performed. Recent reevaluation, three months after stem cell transplant showed complete remission confirmed by bone marrow biopsy and PET-CT scan.

## Discussion

Epidemiology and clinical presentation

BPDCN is a rare disorder arising from blastic plasmacytoid dendritic precursor cells. The incidence is around 0.03-0.04 in 100,000 people although this is probably an underestimation because of the high risk of being misdiagnosed as myelodysplastic syndrome, for example. Because of the rarity of this disease, only a few epidemiological studies have been performed so far [1,2,7]. It has been estimated that BPDCN comprises 0.05% of all hematological malignancies [7]. It is more common in men than women with a ratio of 2-5:1 in older patients/adults. In younger patients, the ratio is more or less equal. There is a bimodal distribution of the incidence adjusted for age, with a peak under 20 and above 60 years of age. The median age at diagnosis ranges from 60 to 70 years [1,2,7]. There is no clear racial predilection, though a recent study in the United States has shown a preference for Caucasians. So far, the reason behind this gender and possible race preference remains unknown [2].

BPDCN was first described in 1994 as a CD4+CD56+ cutaneous lymphoma [8]. The term blastic plasmacytoid dendritic cell neoplasia was first used in 2008. A new category was introduced as part of the acute myeloid leukemia and related neoplasms in the 2008 WHO classification [5]. The fifth edition of the WHO in 2022 classifies BPDCN as part of the histiocytic and dendritic neoplasms [9] whereas in the International Consensus Classification (ICC) 2022, BPDCN is a separate entity within the myeloid neoplasms and acute leukemias [10].

BPDCN can affect multiple organs. The major sites of involvement are the skin, bone marrow, lymph nodes and the central nervous system. Skin lesions occur in 80%-90% with the presence of systemic disease in 50% of cases. Bone marrow with leukemic infiltration is seen in around 60%-90% of cases, lymph node and CNS involvement in, respectively, 40%-50% and 30%-60%. Around 10% of cases present with systemic disease only without skin lesions. The skin lesions are typically reddish-brown or purplish-blue plaques or nodules. These bruise-like nodules often present on sun-exposed skin. Usually skin lesions appear first, followed by other sites getting affected at a later stage [1,11,12].

BPDCN has been associated with other hematological malignancies such as myelodysplastic syndrome, chronic myelomonocytic leukemia, acute myeloid leukemia (AML) and myeloproliferative neoplasms. Concurrence with another hematological malignancy is seen in around 10%-20% of cases [1,13].

Diagnosis

The diagnostic workup to determine the diagnosis and the extent of the disease consists of a biopsy of one or more affected organs (skin, bone marrow, lymph node, etc.), PET scan and lumbar puncture [11]. After taking a biopsy, diagnosis is based on combining morphological, cytochemistry, immunophenotypic, immunohistochemical and genetic findings [12]. According to the WHO, the cells are typically screened for the markers described in Table 4 [9].

**Table 4:** Markers for blastic plasmacytoid dendritic cell neoplasm according to the fifth edition of the WHO classification Immunophenotypic diagnostic criteria of blastic plasmacytoid dendritic cell neoplasm according to the fifth edition of the WHO classification [9].

Expected positive markers	Expected negative markers
CD123	CD3
CD4	CD14
CD56	CD19
TCF4	CD34
TCL1	Lysozyme
CD303	Myeloperoxidase
CD304	

In order to confirm the diagnosis of BPDCN, the following immunophenotypic diagnostic criteria have to be met: expression of CD123 and one other plasmacytoid dendritic cell marker in addition to CD4 and/or CD56 or expression of any three plasmacytoid dendritic cell markers and absent expression of all expected negative markers [9,14]. Morphologically, the cells are blast-like and medium-sized. The karyotype is often complex with multiple chromosomal abnormalities. MYC rearrangement is also frequently present (in 39% of cases), resulting in an immunoblastic variant with higher proliferation and cell growth with a more aggressive course of disease [1,11-13]. Alterations in epigenetic genes such as TET2, ASXL1, SRSF2, IDH2, among others, show an association with other myeloid neoplasms. TP53 mutations occur in 38% of cases [11,12].

Treatment

There is currently no consensus on the optimal treatment strategy for patients with BPDCN. Different options have been proposed such as acute lymphoblastic leukemia (ALL)-based regimens and/or tagraxofusp with allogeneic stem cell transplantation as a consolidating therapy for fit patients. AlloHSCT is the only curative option thus far; without it, most patients will relapse.

Unfit patients are often treated with venetoclax monotherapy or with hypomethylating agents such as azaciditine. Cyclophosphamide, doxorubicin (hydroxydaunorubicin), vincristine (Oncovin) and prednisone (CHOP)-like regimens or AML-based protocols have been used, but responses to these treatments are significantly less good. Treatment regimens based on multiple myeloma protocols have also been used. Bortezomib, lenalidomide, dexamethasone combination therapy (VRd) or daratumumab monotherapy show good responses.

Given the high incidence of CNS involvement in BPDCN, CNS prophylaxis (or treatment in case of proven invasion of the CNS) with intrathecal chemotherapy plays an important role in treating this disease. Studies with combinations of the different options described above are ongoing [1,11,12].

Tagraxofusp is an anti-CD123 antibody, it is a fusion protein combining a diphtheria toxin with recombinant human interleukin 3, which binds to CD123. The toxin is internalized, which leads to cell death. The response rate was around 90% with complete remission in around 75% of patients. The most common adverse effects were capillary leak syndrome, thrombocytopenia and transaminitis [1,11]. Other agents targeting CD123 such as bispecific antibodies and chimeric antigen receptor T cells are currently under investigation [15,16].

ALL-based regimens show better responses than AML-based regimens. The hyper-CVAD regimen (hyper-fractionated cyclophosphamide, vincristine, doxorubicin hydrochloride (adriamycin), and dexamethasone) [6] is most frequently used. An American study showed complete response in 91% of patients treated with the hyper-CVAD protocol [1,11,17].

In order to obtain long-term responses and potentially cure the disease, allogeneic stem cell transplantation is at present the only option. Timing of alloHSCT is important with regards to prognosis. The best results are seen when patients are transplanted in complete remission 1 (CR1) with an overall survival of around 67%. This is in comparison to transplantation in complete remission, where the overall survival is around 8% [11,12,18].

Venetoclax is a BCL2 inhibitor. BCL2 is an anti-apoptotic protein that is also expressed on the leukemic cells in BPDCN. Venetoclax monotherapy is used for patients with BPDCN unfit for intensive chemotherapy and alloHSCT. The same dosing regimen as in the acute myeloid leukemia protocols is applied. Therapy with venetoclax has been proven effective with a duration of response varying from two to 36 months. The most common side effects are cytopenias due to hematologic toxicity and increased risk of infection [11,19].

Prognosis

BPDCN is a rare and aggressive disease with a poor prognosis. The median overall survival in all age groups combined is 12-24 months. The five-year overall survival decreases with increasing age, from 91.5% in patients aged <20 to 26.9% in patients aged >60 years old in an American study. There was no difference in overall survival with respect to race or sex [2]. Thus, it is clear that with aging, the prognosis becomes poorer. Presumably, because younger patients are possible transplant candidates after receiving intensive chemotherapy with a potentially curative intent. Whereas older patients that are no longer transplant eligible are not treated with curative intent and thus have a lower survival rate [17]. Given the lower toxicity profile of tagraxofusp compared to intensive chemotherapy, it may be possible to treat older patients and obtain complete remission in CR1 with the possibility of allogenic stem cell transplant as consolidation. This could improve survival rates for older patients, but further investigations are needed.

## Conclusions

Literature about this rare disease is mostly limited to case reports. No large studies have been conducted and, therefore, there is no consensus yet on the treatment guidelines for BPDCN. Targeted therapies like tagraxofusp are already changing the treatment landscape of BPDCN. But further investigation is needed to determine optimal treatment strategies. Understanding the development of BPDCN, its clinical and pathological features could lead to early and correct diagnosis and new targeted therapies emerging, possibly improving the outcome of this rare and aggressive disease.
